# The influence of the infusion of ephedrine and phenylephrine on the hemodynamic stability after subarachnoid anesthesia in senior adults - a controlled randomized trial

**DOI:** 10.1186/s12871-019-0878-4

**Published:** 2019-11-11

**Authors:** Miodrag Žunić, Nevenka Krčevski Škvarč, Mirt Kamenik

**Affiliations:** Department of Anesthesiology, Intensive Care and Pain Management, University Medical Center Maribor, Ljubljanska ulica 5, 2000 Maribor, Slovenia

**Keywords:** Spinal anesthesia, Hemodynamic stability, Phenylephrine, Ephedrine

## Abstract

**Background:**

We studied the influence of ephedrine or phenylephrine infusion administered immediately after spinal anesthesia (SA) on hemodynamics in elderly orthopedic patients.

**Methods:**

A prospective, randomized, double-blind, placebo-controlled study.

After a subarachnoid injection of 15 mg of levobupivacaine, the participants received an infusion of either ephedrine 20 mg (E group), phenylephrine 250 mcg (P group) or saline (C group) within 30 min. We measured blood pressure, cardiac index (CI) and heart rate (HR) from 15 min before to 30 min after SA.

**Results:**

Seventy patients were included in the final analysis. At the end of measurements, mean arterial pressure (MAP) decreased significantly after SA in comparison to the baseline value in the C group but was maintained in the P and E group, with no significant differences between the groups. CI decreased after SA in the C group, was maintained in the P group, and increased significantly in the E group with significant differences between the C and E group (*p* = 0.049) also between the P and E (*p* = 0.01) group at the end of measurements. HR decreased significantly after SA in the C and P group but was maintained in the E group, with significant differences between the P and E group (*p* = 0.033) at the end of measurements.

**Conclusions:**

Hemodynamic changes after SA in elderly orthopedic patients can be prevented by an immediate infusion of phenylephrine or ephedrine. In addition to maintaining blood pressure, the ephedrine infusion also maintains HR and increases CI after SA.

**Trial registration:**

ISRCTN registry with registration number ISRCTN44377602, retrospectively registered on 15 June 2017.

## Background

Many European countries’, England’s, Australia’s and Canada’s national arthroplasty registers have showed an increasing of the prevalence of the hip and knee arthroplasty over the last decades [1–4]. The number of such procedures in the future seems to be even higher [3]. Many of those are elderly patients [1].

A commonly used technique for elderly patients undergoing orthopedic surgery is spinal anesthesia (SA) [5, 6]. Hypotension is a side effect often associated with SA [7]. The incidence of SA-induced hypotension (SAIH) in the elderly, which has been estimated as high as 80% [8] is due to a decrease of the systemic vascular resistance (SVR) and cardiac output (CO) [5]. The high incidence of comorbid conditions in the elderly leads to high risk for hypo perfusion caused by hypotension, [5, 9, 10], which main risk factor is hypovolemia [11, 12]. The administration of crystalloids can quickly lead to volume overload and signs of congestive heart failure in the elderly and is often not effective in maintaining blood pressure [5]. Therefore, ephedrine and phenylephrine are the vasopressors of choice for the prevention of SAIH in the elderly [13].

The objective of our study was to evaluate the effectiveness of prophylactic intravenous (IV) ephedrine or phenylephrine infusion on the prevention of hypotension and a decrease in CO following SA in patients older than 60 years undergoing elective orthopedic surgery.

## Methods

Our prospective, randomized, double-blind, placebo-controlled study was approved by the National Medical Ethics Committee at the Ministry of Health, Republic of Slovenia (protocol number 0120–8/2017–3, KME 21/01/17. The trial was retrospectively registered at ISRCTN with the submission number ISRCTN44377602. Our study adheres to CONSORT guidelines.

We studied 84 patients older than 60 years, scheduled for orthopedic hip or knee replacement surgery under SA. Written informed consent was obtained from each patient. Participant exclusion criteria were any contraindications to SA (absolute: patients’ refusal, infection at the site of injection, uncorrected hypovolemia, allergy, increased intracranial pressure or relative: coagulopathy, sepsis, fixed CO states, indeterminate neurological disease) or administration of vasoconstrictors (allergy or hypersensitivity to vasoconstrictors, unstable angina, recent coronary artery bypass surgery, recent myocardial infarction refractory arrhythmias, untreated or uncontrolled severe hypertension, untreated or uncontrolled heart failure, uncontrolled hypothyroidism, sulfite sensitivity, uncontrolled diabetes, pheochromocytoma, use of cocaine, monoamine oxidase inhibitors, phenothiazine compounds or tricyclic antidepressants).

Patients were fasted the night before the surgery. Antihypertensive medications were discontinued the day before the surgery, except the β-blockers. Midazolam 7.5 mg was given for premedication one hour before the surgery. After the patient arrival in the operating room, an IV line was inserted and the patient was placed in the lateral decubitus position. Using the paramedian approach with a 25-gauge Sprotte needle (PAJUNK, GmbH, Geisingen, Germany) we performed lumbar puncture at the L2–L3 interspace. With the needle aperture oriented in the cephalad direction, 3 mL of 0.5% levobupivacaine (Chirocaine 0.5% plain; AbbVie, Campoverde, Italia) were injected within 15 s.

**Fig. 1:**
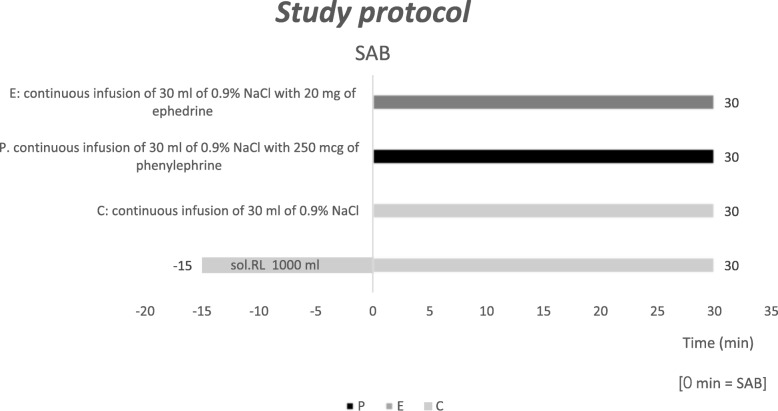
Study protocol: Fluid flow-chart

All the patients received an infusion of 1000 mL lactated Ringer solution within 45 min from the beginning of the measurement (500 ml before and 500 ml after SA) by opening the IV roller clamp and “eyeballing” the infusion rate. With respect to the infusion of treatment medication, the patients were randomly assigned to one of the three groups using sealed envelope randomization. The C group (control group) received an infusion of 30 ml 0.9% NaCl 30 min after SA. The P group (phenylephrine group) received a continuous infusion of 30 ml of 0.9% NaCl with 250 mcg of phenylephrine 30 min after SA. The E group (ephedrine group) received a continuous infusion 30 ml of 0.9% NaCl with 20 mg of ephedrine 30 min after SA. The infusion of the treatment medication in all the groups was started immediately after SA (Fig. 1) via a volumetric IV pump (Alaris™ GH model 80023xx01, CareFusion).

We measured non-invasive blood pressure, non-invasive CO using thoracic electrical bioimpedance (TEB) method, heart rate and pulse oximetry (SpO_2_) using the AESCULON, OSYPCA MEDICAL, 2011, monitor.

We started hemodynamic measurements 5 min after placing the patient in the lateral decubitus position and we recorded it for 45 min (for 15 min before and 30 min after the injection of the local anesthetic solution into the subarachnoid space). Data on blood pressure were recorded and stored on the hard drive at 5-min intervals and other hemodynamic data at 1-min intervals.

The protocol for rescue treatment in the event of hemodynamic instability included:

1. Severe hypotension (decrease of systolic blood pressure for more than 30% from baseline, or systolic blood pressure less than 80 mmHg): additional ephedrine boluses of 5 mg repeated in 3 min with additional infusion of Ringer solution or additional phenylephrine boluses of 50 mcg repeated in 3 min with additional infusion of Ringer solution.

2. Bradycardia (≤50 beats per minute): bolus of atropine 0.5 mg, repeated in 1 min until heart rate frequency is more than 50 beats per minute or overall amount of 2 mg atropine is reached.

3. Hypertension (increase of systolic blood pressure for more than 30% from baseline): discontinuation of the ongoing infusion.

Hypotensive, hypertensive or bradycardic patients were defined as patients who developed at least one episode of hypertension, hypotension or bradycardia throughout the case and were treated according to the protocol.

Data were analyzed with the SPSS 25.0.0. software. Demographic data and baseline values were compared with one-way analysis of variance and χ^2^, where appropriate. The hemodynamic data before, 10, 20 and 30 min after SA were compared with ANOVA with Bonferroni correction for post hoc comparisons and the Student’s t-test for paired samples, where appropriate. In addition, the analysis of variance for repeated measurements with Bonferroni correction was performed to compare the change in hemodynamic measurements between the three treatment groups and the change over time. *P* < 0.05 was considered statistically significant.

Sample size calculation to detect a 20% difference in CI (0.5 L/min/m^2^) between treatment groups, assuming a mean CI of 2.5 L/min/m^2^ and SD of 0.5 L/min/m^2^ with a probability level of 0.05 and a power of 0.85, yielded a sample size of 69 patients. The same number of patients would detect a 10 mmHg difference between the groups in MAP (assuming SD of 10 mmHg) with a probability level of 0.05 and a power of 0.85. Expecting dropouts due to various reasons, including side effects, we randomized 90 patients. We used the G Power 3.1.9.2. software for these calculations.

## Results

**Fig. 2:**
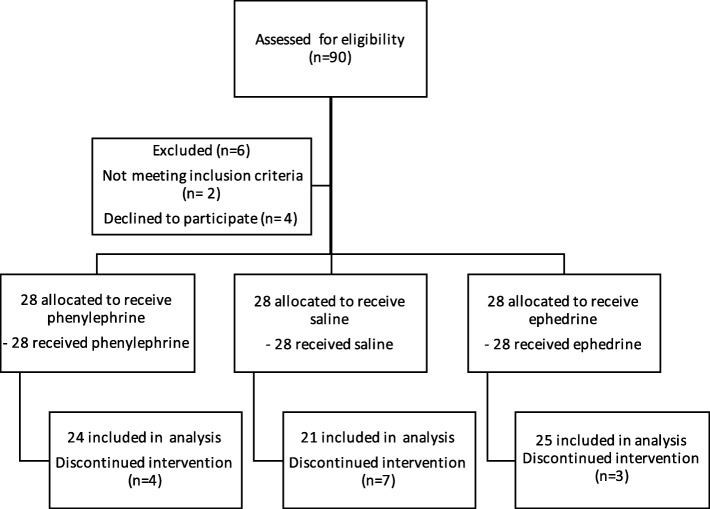
Randomization and follow-up of the patients

We randomized 90 patients. Four patients were excluded because they declined to participate and two patients were excluded for not meeting the inclusion criteria (Fig. 2). Patients with side effects requiring a rescue protocol were excluded from final analysis and were analyzed separately. Seven patients from the C group were excluded because of hemodynamic instability (5 patients got severe hypotension, one had bradycardia and one had hypotension with bradycardia). Two patients were excluded from the P group because of hypotension with bradycardia and two had hypotension episode alone. Two patients were excluded from the E group because of hypotension, one patient because of bradycardia and hypotension. Therefore, 70 patients were included in the final analysis: Twenty-five patients in the E group, twenty-four in the P group and twenty-one patients in the C group.

There were no significant differences between the groups with respect to the demographic data and baseline hemodynamics (Table 1).

**Table 1:** Demographic data and baseline hemodynamic measurements in the three groups of patients

GROUP	C	P	E	*p* value
Number of patients	21	24	25	
Gender (male/female)	5/16	8/16	5/20	0.55
Age (years)	67.9 (± 5.5)	71.1 (± 7.7)	68.4 (± 6.6)	0.209
Height (cm)	166.2 (± 8.2)	158.8 (± 34.6)	164.6 (± 11.5)	0.477
Weight (kg)	79.7 (± 13.5)	86.0 (± 13.6)	82.1 (± 14.2)	0.305
ASA I/ ASA II/ ASA III	3/12/6	2/15/7	1/17/7	0.803
BMI	28.8 (± 4.5)	31.3 (± 5.0)	30.2 (± 3.9)	0.195
BSA	1.9 (± 0.2)	1.9 (± 0.2)	1.9 (± 0.2)	0.54
SBP (mmHg)^a^	134 (± 22)	128 (± 17)	125 (± 15)	0.194
MAP (mmHg)^a^	90 (± 12)	85 (± 12)	84 (± 9)	0.156
HR (beats/min) ^a^	75.4 (± 11.0)	73.5 (± 11.6)	74.7 (± 11.7)	0.856
CI (BSA) (l/min/m^2^) ^a^	2.5 (± 0.7)	2.2 (± 0.5)	2.5 (± 0.6)	0.219
SVR (din s cm^− 5^) ^a^	886 (± 292)	863 (± 233)	850 (± 320)	0.911
SVI (ml/m^2^) ^a^	33.1 (± 5.8)	30.3 (± 6.0)	32.8 (± 6.7)	0.255

*SBP*systolic blood pressure, *MAP*mean arterial pressure, *CO* cardiac output,

*SVR*systemic vascular resistance

Values are mean (± SD)

^a^ Baseline values defined as last registered values before SA

**Fig. 3:**
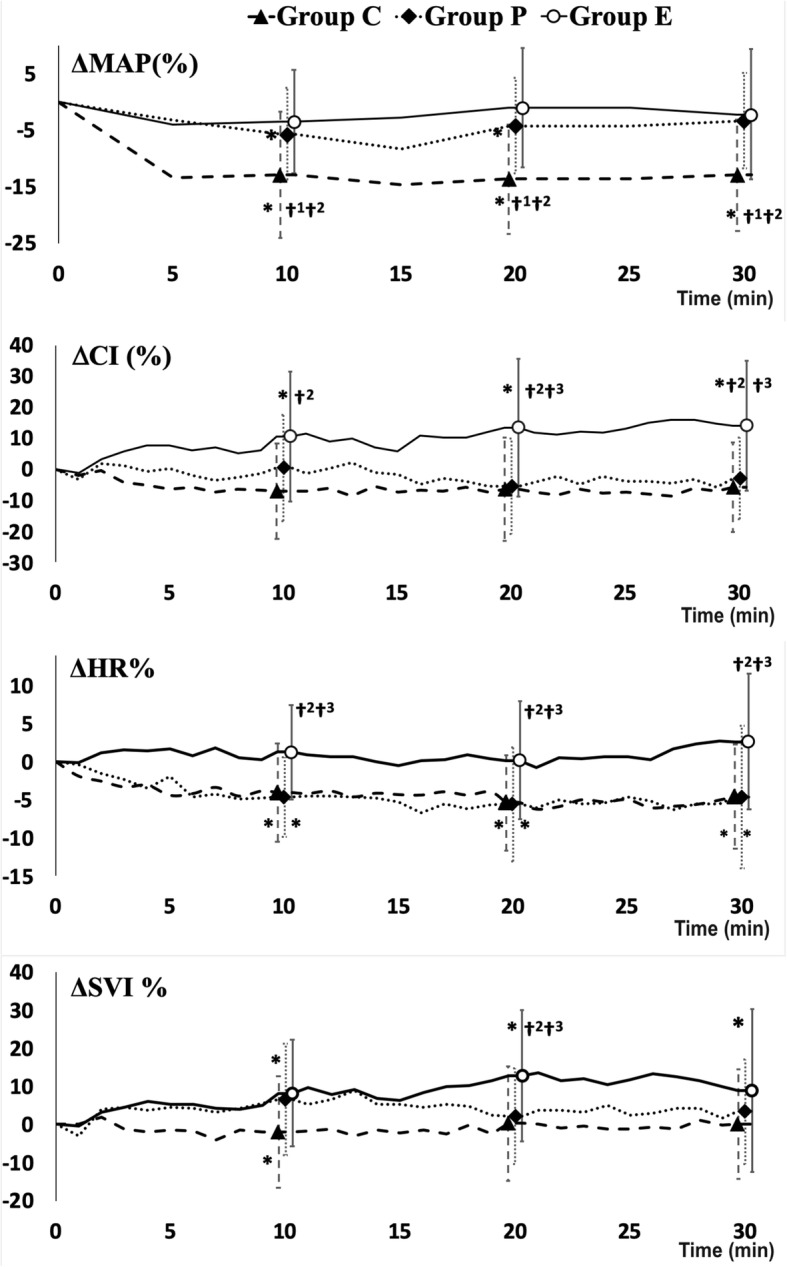
The change of mean arterial pressure (MAP), heart rate (HR), cardiac index (CI), and stroke volume index (SVI) from the baseline till 30 min after SA in three groups of patients (Group C = control group; Group P = phenylephrine group; Group E = ephedrine group). * *P* < 0.05 with respect to baseline, †^1^ *P* < 0.05 between the Group P and Group C, †^2^ *P *< 0.05 between the Group E and Group C, †^3^ *P* < 0.05 between the Group P and Group E

The change in hemodynamic data after spinal block in the three treatment groups is shown in Fig. 3. The decrease in MAP after spinal block was most prominent in the C group and was statistically significant 10, 20 and 30 min after the block. In the P group a transient statistically significant decrease in MAP was measured 10 (*p* = 0.002) and 20 (*p* = 0.02) minutes after the block, but by the end of measurements MAP returned to about baseline values. MAP was maintained after SA in the E group. At the end of measurements, the decrease in MAP was significantly higher in the C group compared to the E (*p* = 0.004) and P group (*p* = 0.043), but there were no differences between the P and E group (Fig. 3).

CI after SA decreased non-significantly in the C and P group (Fig. 3). In the E group, CI significantly increased after SA. At the end of measurements, CI was significantly increased in the E group in comparison to the C (p<0.001) and P group (*p* = 0.002), with no differences between the C and P group. HR decreased significantly after SA in the C and P group but not in the E group (Fig. 3). At the end of measurements, the decrease of HR after SA was significantly higher in the C (*p* = 0.017) and P group (*p* = 0.013) in comparison to the E group, with no significant differences between the C and P group (Fig. 3). SVI did not change after SA in the C group, transiently increased (10. minutes after the block) in the P group (*p* = 0.036) and increased significantly in the E group (*p* = 0.049) [Fig. 3]. 20 min after SA the increase in SVI was significantly higher in the E group in comparison with the C (*p* = 0.02) and P group (*p* = 0.48) [Fig. 3].

Fourteen patients experienced side effects (Table 2). There were no significant differences between groups. The incidence of bradycardic and hypotensive events (*n* = 14) was not statistically different between the treatment groups (*p* = 0.33). Although the number of the bradycardic (*n* = 3) and the hypotensive (*n* = 6) patients was higher in the group C, the differences between the groups did not reach statistical significance (*p* = 0.86 and *p* = 0.53). There were no significant differences between the groups with respect to the number of patients receiving ephedrine (*p* = 0.58) or phenylephrine (*p* = 0.54) as the rescue drug.

**Table 2:** Number of patients developing side effects, time to onset of side effect and rescue medication in each treatment group. Seven patients developed hypotension and bradycardia

GROUP	C	P	E
BRADYCARDIA			
Number of patients	3/28	2/28	2/28
Time to event (min)	26.7 ± 8.1	23.5 ± 2.1	27.5 ± 17.2
Dose of atropine (mg)	0,7 ± 0.3	0.5 ± 0	0.5 ± 0
HYPOTENSION			
Number of patients	6/28	4/28	3/28
Time to event (min)	25.8 ± 6.7	27.3 ± 5.6	27 ± 7.5
Ephedrine			
Number of patients	3/28	1/28	2/28
Dose of ephedrine ^a^ (mg)	11.7 (10–15)	10	10 (10)
Phenylephrine			
Number of patients	3/28	3/28	1/28
Dose of phenylephrine ^a^ (mg)	100 (100)	167 (100–300)	100 (100)

## Discussion

We studied the influence of prophylactic treatment with two different vasopressors (ephedrine and phenylephrine) on the prevention of hypotension after SA and the changes on hemodynamics in the elderly patients. The topic is undoubtedly very important and to our knowledge, our study is the first, which documented the efficacy of a prophylactic ephedrine infusion after SA in elderly. Many studies have showed the importance of prevention of even brief periods of hypotension in the elderly patients, which could avoid complications and mortality [14–16].

All the patients in our study were receiving a continuous infusion of Ringer’s solution during the time of measurements. The study showed that the additional infusion of ephedrine maintained the MAP after SA (E group). MAP was also maintained in the P group, but the additional infusion of phenylephrine was not as effective as the ephedrine infusion. In patients not receiving vasopressors (C group), MAP decreased after SA by about 14%. CI was maintained after SA in the C and P group and increased in the E group by about 14%. HR decreased after SA in the C and P group and was maintained in the E group.

Nakasuji et al. showed a decrease in SVR, not CO, is the main mechanism of hypotension seen during SA in elderly patients [17]. Marhofer and co-workers [18] also showed that SAIH in elderly ASA 3 patients was caused by a decrease in systemic vascular resistance index, without change in cardiac index. Kamenik and Eržen [19] showed that CO decreases after SA in the group of middle-aged patients not receiving crystalloids as well as in the group of patients who received crystalloids before SA, while CO increases in the group of patients receiving lactated Ringer’s solution at the time of spinal block. Later on, Zorko and Kamenik [20] have shown that the infusion of 1000 mL of lactated Ringer after spinal block prevented the decrease of CO after SA, with CO actually increasing while the infusion was running. In our study, we used a slightly modified hydration strategy. We infused 1000 ml of Ringers solution continuously during the period of measurements (15 min before and 30 min after SA). Our results show that with this regimen we were able to maintain CI after SA at about baseline values in the C group. However, in this group of patients we were not able to maintain the MAP, which presumably decreased because of the decreased systemic vascular resistance. In addition, HR also decreased after SA in the C group. Additional infusion of the pure α (alpha) vasoconstrictor phenylephrine blunted most of the decrease in MAP. However, the infusion of phenylephrine did not prevent the decrease in HR after SA. In the study of Stewart et al., the authors found a dose-dependent reduction in both maternal HR and CO, measured with suprasternal Doppler, when comparing three different infusion regimens of phenylephrine. The highest infusion rate reduced both CO and HR by > 20% [21]. In our study in the P group, HR also decreased, but due to a slight increase in SVI, CI was maintained. The slight increase in SVI in the P group in comparison with the C group was probably caused by the action of the vasopressor on the venous tone, since the volume of infusion was the same in both groups. In the E group with the infusion of ephedrine, we were able to maintain the MAP while the HR and SVI increased causing an increase in the CI. An additional increase in HR and SVI in the E group were presumably due to the inotropic action of ephedrine. The incidence of patients with side effects was higher in the C group (*n* = 7) in comparison with the P group (*n* = 4 and the E group (*n* = 3). Although the differences were not statistically significant (due to a low sample size), a nearly double incidence of side effects was found in C group. Since the main interest in our study was hemodynamics of vasopressors, we decided to exclude the patients with side effects requiring rescue drugs in the final data presentation. We could also consider a lower dose of local anesthetic approach for intrathecal anesthesia for the elderly, in order to minimize the side effects.

The best vasopressor may in fact be different, depending on the patient population. The current opinion in obstetrics favors the use of phenylephrine over ephedrine as alpha agonist of choice for treating hypotension associated with SA due to its beneficial effect on fetal acid-base balance and umbilical pH [22, 23]. Mon and coworkers have shown that ephedrine infusion was associated with good systolic blood pressure control and no reduction in both the maternal CO and HR, than those in phenylephrine group [24]. On the other hand, Larson et al. [25] reviewed that many researchers suggest phenylephrine may adversely affect cerebral oxygen saturation and perfusion. The authors stated that the use of phenylephrine to treat SAIH is a ubiquitous practice among anesthesia providers, despite the fact that it has never been shown to improve outcomes. Another approach could be the combined use of the vasopressors and different timings, but according to Das et al. [26], in obstetric patients such combination of half the usual dose of ephedrine and phenylephrine had no supreme outcome.

Regardless of prehydration, a high incidence of hypotension (the overall incidence of SAIH was 49%, ranging from 39% in the colloid group to 62% in the crystalloid group) follows SA in normovolemic elderly patients undergoing elective procedures [27]. Our study shows the importance of prophylactic infusion treatment with vasopressors in this group of patients, but a definite and widely available method of predicting pre-operative hypotension is not identified yet. Berlac and Rasmunssen suggested that NIRS could provide an early warning of hypotension [28]. On the other hand, Hanss et al. reported that women who developed more severe hypotension after SA had greater changes in heart rate variability [29].

In our study, CO was measured with TEB, noninvasively. Most researchers have accepted the TEB method for monitoring the trends of change in CO, but the absolute value of CO measured with TEB is controversial [30–32]. As a non-invasive method in spontaneously breathing patients during SA, it has an advantage over invasive methods, which are ethically rarely justified [33]. In our study, the TEB method was used also as a trend monitor to follow the changes in hemodynamic parameters after SA.

## Conclusion

In conclusion, our study shows that we can preserve MAP after SA with the combination of the Ringers solution infusion with the infusion of ephedrine or an infusion of phenylephrine. An ephedrine infusion also prevents a decrease in HR and increases cardiac output, while an infusion of phenylephrine maintains CO, but is accompanied by a decrease in HR. Since flow is better maintained, we recommend the infusion of ephedrine in elderly patients receiving SA. Further studies are necessary to evaluate the duration of the effects of the infusion of ephedrine and the optimal dose to improve hemodynamic stability after SA in the elderly.

## Data Availability

The datasets generated and/or analyzed during the current study are available from the corresponding author on reasonable request.

## Abbreviations

ANOVA: Analysis of variance
ASA: the *American Society of Anesthesiologists*
C group: Control group
CI: Cardiac index
CO: Cardiac output
E group: Ephedrine group
HR: Heart rate
IV: intravenous
MAP: Mean arterial pressure
NIRS: Near-infrared spectroscopy
P group: Phenylephrine group
pH: Power of hydrogen
SA: Spinal anesthesia
SAIH: Spinal anesthesia induced hypotension
SAP: Systolic arterial pressure
SD: Standard deviation
SPSS: Statistical package for the social sciences
SV: Stroke volume
SVI: Stroke volume index
SVR: Systemic vascular resistance
TEB: Thoracic electrical bioimpedancy
